# Interfacial Redox Regulation of γ-Al_2_O_3_-Supported MnFe_2_O_4_ for Oxidative Chain Scission in Concentrated Poly(vinyl alcohol) Gel-Forming Networks

**DOI:** 10.3390/gels12090848

**Published:** 2026-09-17

**Authors:** Zhong Pan, Qilong Sun, Xiuyu Shen, Yanyan Li, Xiaoyun Long, Shuang Zhai

**Affiliations:** 1 School of Textile and Clothing, Nantong University, Nantong 226019, China; thezhong1111@163.com (Z.P.); sunqilong001@ntu.edu.cn (Q.S.); 2 School of Textile Science and Engineering, Shaoxing University, Shaoxing 312000, China; 2024000012@usx.edu.cn; 3 College of Material and Textile Engineering, Jiaxing University, Jiaxing 314001, China; liyan2922@126.com

**Keywords:** Poly(vinyl alcohol), PVA gel-forming networks, oxidative chain scission, interfacial redox regulation, heterogeneous Fenton-like catalysis

## Abstract

Concentrated poly(vinyl alcohol) (PVA) gel-forming networks are characterized by extensive hydrogen bonding, pronounced chain entanglement, and restricted molecular diffusion, which collectively limit oxidant accessibility and impede efficient oxidative transformation. Herein, a γ-Al_2_O_3_-supported MnFe_2_O_4_ catalyst (FMA) was developed to regulate interfacial redox properties and enhance H_2_O_2_ activation, thereby promoting oxidative chain scission of concentrated PVA gel-forming networks. The γ-Al_2_O_3_ support effectively suppressed MnFe_2_O_4_ aggregation, leading to enhanced specific surface area and increased exposure of accessible Fe/Mn active sites. Under optimized conditions, the FMA/H_2_O_2_ system achieved approximately 90% PVA conversion within 120 min, together with 89.2% TOC removal and a decrease in molecular weight from 68,892 to 2063 Da, demonstrating efficient oxidative chain scission and deep mineralization. Mechanistic investigations using XPS, EPR, and radical-quenching experiments revealed that synergistic Fe/Mn redox cycling promoted H_2_O_2_ activation and hydroxyl radical generation, which dominated PVA chain cleavage. The catalyst exhibited excellent stability with limited metal leaching during repeated cycles. This work demonstrates that γ-Al_2_O_3_-mediated interfacial regulation provides an effective strategy for designing supported metal oxide catalysts toward controlled transformation of highly entangled gel-related polymer networks, providing insights into the oxidative deconstruction of PVA-based gel and hydrogel systems.

## 1. Introduction

Poly(vinyl alcohol) (PVA) is a representative water-soluble polymer widely used in hydrogel and polymer-network systems owing to its excellent film-forming ability, abundant hydroxyl groups, and favorable physicochemical stability [1,2,3,4]. Its carbon backbone and extensive intra- and intermolecular hydrogen-bond interactions contribute to the structural stability of PVA in aqueous environments [3,5,6,7,8]. At elevated concentrations, PVA solutions exhibit high viscosity, pronounced chain entanglement, and restricted molecular diffusion, resulting in a transport-limited polymer environment where interactions among oxidants, reactive species, catalytic interfaces, and polymer chains are significantly restricted [2,5,6,7]. In addition, the physicochemical properties of concentrated polymeric and hydrogel-like networks play a critical role in regulating molecular transport processes. Factors such as the water-to-polymer ratio, polymer volume fraction, network density, chain entanglement, and connectivity collectively determine molecular transport behavior within polymer matrices [9,10,11]. In concentrated PVA systems, these transport limitations are further associated with the formation of physically cross-linked hydrogel-like networks. The PVA system used in this study (100 g L^−1^, approximately 10 wt%) is above the reported critical entanglement concentration of approximately 4.4 wt%, indicating the formation of an entangled polymer network. At a comparable PVA concentration, intermolecular hydrogen bonding between hydroxyl groups has been reported to generate a physically cross-linked network with a characteristic mesh size (ξH) of approximately 554 nm, containing approximately 22 PVA chains per mesh [9]. Such interconnected structures increase chain connectivity and reduce the available free volume for molecular diffusion. Moreover, the water-to-polymer ratio (~9:1 in the 10 wt% PVA system) determines the accessibility of solvent molecules and reactants within the polymer matrix [12]. Consequently, efficient chemical transformation of concentrated PVA requires not only highly reactive catalytic centers but also improved accessibility of catalytic interfaces and effective utilization of interfacial active sites [6,13,14].

H_2_O_2_-driven advanced oxidation processes, particularly Fenton and Fenton-like reactions, provide an effective pathway for oxidative polymer transformation through the generation of reactive oxygen species (ROS) [15,16,17], which can directly attack polymer backbones and induce oxidative chain scission [18]. The activation efficiency of oxidants is a key factor determining the performance of heterogeneous oxidation processes. In catalytic oxidation systems, the interaction between oxidants and catalytic interfaces, rather than the intrinsic oxidation capability of metal species alone, plays a crucial role in regulating reactive species generation. Recent studies have demonstrated that modulation of metal oxidation states, interfacial electron-transfer pathways, and surface oxygen environments can effectively enhance oxidant activation and improve catalytic oxidation efficiency [19,20,21]. For example, the construction of multifunctional metal-support interfaces can optimize the local electronic structure of active centers and promote redox cycling, thereby facilitating continuous oxidant conversion and reactive species production. However, conventional homogeneous Fenton systems are limited by narrow operational pH windows, iron-sludge generation, and inefficient regeneration of Fe active species [16,17,22,23]. Heterogeneous Fenton-like catalysts provide an alternative by immobilizing redox-active centers on solid interfaces, thereby enabling catalyst recovery and sustained interfacial H_2_O_2_ activation [13,17,24,25].

Manganese ferrite (MnFe_2_O_4_) has attracted considerable attention as a heterogeneous Fenton-like catalyst because of its magnetic separability and the multiple accessible oxidation states of Fe and Mn species [13,14,24,25,26]. The coexistence of Fe and Mn centers enables a coupled redox network, in which Mn species can participate in interfacial electron-transfer processes and facilitate the regeneration of Fe^2+^ during H_2_O_2_ activation [14,27,28,29]. Such multivalent redox chemistry is favorable for sustaining reactive oxygen species generation and subsequent oxidative chain conversion. However, bare MnFe_2_O_4_ nanoparticles are prone to severe aggregation because of magnetic interactions and high surface energy, which decreases the exposure of Fe/Mn active sites and restricts interfacial contact with reactants [13,30,31]. This limitation becomes particularly important in concentrated polymer environments, where molecular transport is already restricted. Therefore, constructing an appropriate support interface to regulate nanoparticle dispersion, active-site accessibility, and interfacial redox utilization represents an effective strategy for improving MnFe_2_O_4_-based H_2_O_2_ activation [13,14,32].

γ-Al_2_O_3_ is widely used as a catalyst support because of its porous structure, thermal stability, and abundant surface hydroxyl groups [32,33,34,35,36]. In particular, surface hydroxyl groups can provide anchoring sites for metal precursor species, thereby restricting their migration and aggregation during subsequent thermal conversion [33,35,37]. These metal–support interactions can regulate nanoparticle dispersion, alter the local surface oxygen environment, and improve the accessibility of interfacial active sites [35,37,38,39,40,41]. Accordingly, γ-Al_2_O_3_ may function not merely as a high-surface-area support, but also as an interfacial regulator that modulates the spatial distribution and local chemical environment of Fe/Mn redox centers. Although γ-Al_2_O_3_-supported Fe-based catalysts have been investigated in heterogeneous oxidation reactions [32,35,40], the role of γ-Al_2_O_3_-mediated interfacial regulation in MnFe_2_O_4_ systems, particularly its relationship with active-site accessibility, interfacial redox behavior, and oxidative chain scission of concentrated PVA, remains insufficiently understood.

**Scheme 1:**
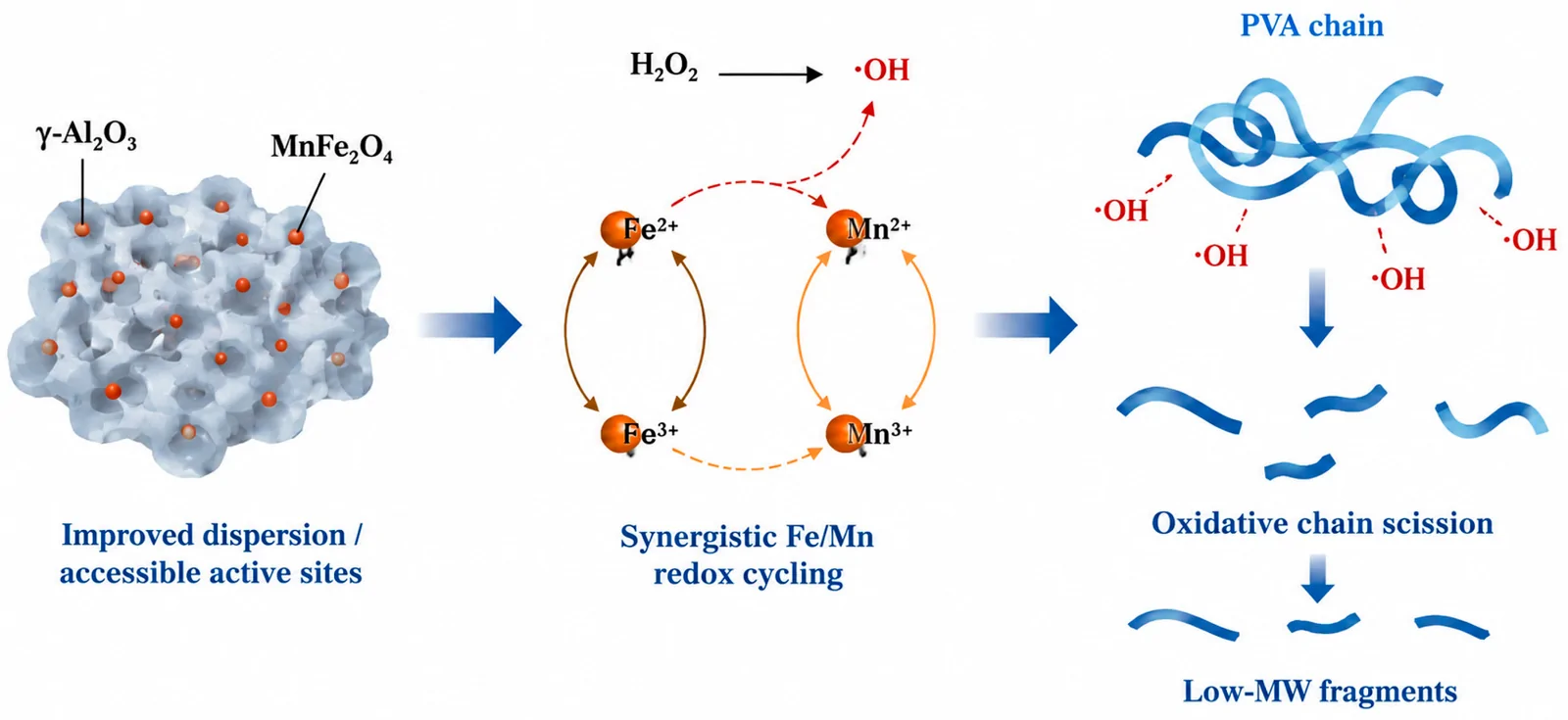
Schematic illustration of H_2_O_2_ activation and oxidative chain scission of PVA over γ-Al_2_O_3_-supported MnFe_2_O_4_ through synergistic Fe/Mn redox cycling.

Therefore, this study establishes an effective strategy for γ-Al_2_O_3_-mediated interfacial regulation of MnFe_2_O_4_. As illustrated in Scheme 1, γ-Al_2_O_3_ modulates the dispersion of MnFe_2_O_4_ nanoparticles and increases the exposure of Fe/Mn active sites. The exposed active sites further promote H_2_O_2_ activation via synergistic Fe-Mn redox cycling, thereby accelerating the oxidative chain scission of concentrated PVA gel-forming networks. By correlating interfacial structure with H_2_O_2_ activation and polymer-chain transformation, this study aims to provide mechanistic insight into the design of multifunctional supported transition-metal oxide interfaces for efficient redox conversion of concentrated, transport-restricted polymer systems, with particular relevance to the oxidative deconstruction of PVA gel-forming and hydrogel-related networks.

## 2. Results and Discussion

### 2.1. Structural and Interfacial Regulation of MnFe_2_O_4_ by γ-Al_2_O_3_

To establish the relationship between catalyst structure, interfacial redox properties, and PVA oxidative conversion, a combination of structural, surface, electrochemical, and reactive-species analyses was employed. SEM and TEM were used to examine catalyst morphology, nanoparticle dispersion, and interfacial structure, while XRD was used to identify the crystalline phases and structural integrity of MnFe_2_O_4_ after loading on γ-Al_2_O_3_. N_2_ adsorption–desorption analysis was performed to evaluate the surface area, pore characteristics, and accessibility of the supported catalyst. XPS and FTIR were used to probe the surface chemical states and interfacial interactions, whereas electrochemical measurements were employed to assess interfacial charge-transfer behavior. EPR spectroscopy and radical-quenching experiments were further used to identify the reactive oxygen species involved in H_2_O_2_ activation. These analyses were combined with PVA conversion and molecular-weight measurements to correlate catalyst structure and interfacial redox behavior with oxidative chain scission.

Given that polymer-network structure can also govern molecular transport and catalytic accessibility, the physical state of the 100 g L^−1^ PVA system was further considered in the context of its reported rheological behavior. Previous rheological studies have shown that 10 wt% PVA, well above the reported critical entanglement concentration (~4.4 wt%), exhibits weak-gel-like behavior characterized by a low-frequency G′ plateau and G′ > G″, arising from transient intermolecular hydrogen-bonded networks [12]. Therefore, the term “gel-forming PVA network” herein refers to a highly entangled, physically associated PVA state rather than a permanently crosslinked hydrogel.

Prior to detailed structural characterization, the bulk elemental composition of FMA was quantitatively determined by ICP-OES to verify the actual incorporation of Fe and Mn species during catalyst preparation. As summarized in Table 1, the measured Mn and Fe contents in FMA were 4.43 wt.% and 9.42 wt.%, respectively, corresponding to an experimental Fe/Mn molar ratio of 2.12:1. This value is close to the theoretical ratio of MnFe_2_O_4_ (2:1), indicating that the designed Fe–Mn composition was successfully achieved during synthesis. The confirmed Fe/Mn stoichiometry provides a reliable foundation for subsequent investigation of the interfacial redox properties and catalytic performance of FMA.

**Figure 1:**
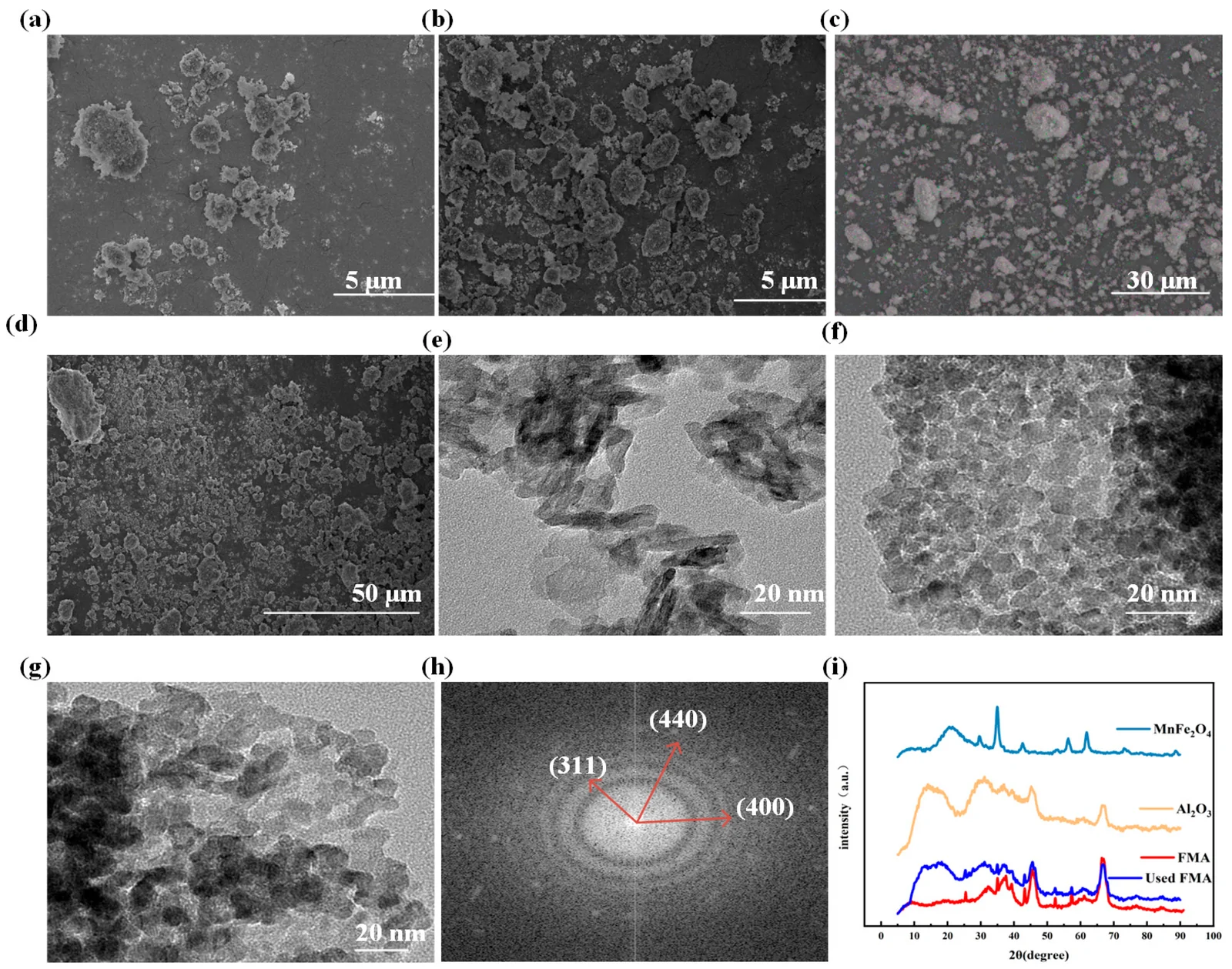
SEM images of (**a**) Al_2_O_3_, (**b**) MnFe_2_O_4_, (**c**) FMA, and (**d**) used FMA after four catalytic cycles; TEM images of (**e**) Al_2_O_3_, (**f**) FMA, and (**g**) used FMA after four catalytic cycles; (**h**) FFT pattern of FMA; and (**i**) XRD patterns of Al_2_O_3_, MnFe_2_O_4_, FMA, and used FMA.

The morphology and dispersion characteristics of the catalysts were investigated by SEM and TEM. As shown in Figure 1a,b, pristine γ-Al_2_O_3_ exhibits an irregular porous morphology, whereas bare MnFe_2_O_4_ displays evident particle aggregation caused by intrinsic magnetic interactions and the high surface energy of ferrite nanoparticles [13,30,31]. Such aggregation decreases the exposure of surface Fe/Mn sites and restricts reactant accessibility to catalytic interfaces. After introducing γ-Al_2_O_3_, the FMA catalyst presents a distinctly different morphology (Figure 1c,d). MnFe_2_O_4_nanoparticles are uniformly distributed on the γ-Al_2_O_3_ surface without obvious large agglomerated domains, indicating that the support effectively suppresses nanoparticle migration and aggregation during calcination [30,35]. The improved dispersion suggests that γ-Al_2_O_3_ provides anchoring interfaces for Fe/Mn precursor species and promotes the formation of highly accessible catalytic domains [33,35,37].

Therefore, γ-Al_2_O_3_ plays an active role in regulating the interfacial structure of MnFe_2_O_4_ rather than acting solely as an inert support. TEM observations further reveal the microstructural differences between MnFe_2_O_4_ and FMA. As shown in Figure 1e, γ-Al_2_O_3_ exhibits a porous framework with irregular contrast distribution. After loading MnFe_2_O_4_, numerous nanosized particles are uniformly dispersed on the γ-Al_2_O_3_ surface (Figure 1f), confirming the successful construction of the supported composite structure. High-resolution TEM analysis (Figure 1g) shows clear lattice fringes with interplanar spacings of approximately 0.254, 0.213, and 0.150 nm, corresponding to the (311), (400), and (440) planes of cubic spinel MnFe_2_O_4_, respectively [13,25,42]. No obvious lattice distortion or secondary crystalline phase was detected, indicating that the spinel structure of MnFe_2_O_4_ was preserved after loading onto γ-Al_2_O_3_. Particle-size analysis based on TEM images shows that the average particle size decreases from approximately 35.6 nm for MnFe_2_O_4_ to 12.7 nm for FMA, further confirming the dispersion-regulation effect of γ-Al_2_O_3_.

The crystalline structures of the catalysts were analyzed by XRD (Figure 1h). Bare MnFe_2_O_4_ exhibits characteristic diffraction peaks located at approximately 30.2°, 35.5°, 43.1°, 53.5°, 57.0°, and 62.6°, corresponding to the (220), (311), (400), (422), (511), and (440) planes of cubic spinel MnFe_2_O_4_ [13,25,42]. The characteristic diffraction peaks of MnFe_2_O_4_ remained unchanged after loading, indicating that the spinel crystalline phase was retained during the impregnation–calcination process. However, the diffraction intensity of MnFe_2_O_4_ in FMA is significantly weakened compared with bare MnFe_2_O_4_, which can be attributed to the reduced crystallite size and improved dispersion of MnFe_2_O_4_ nanoparticles on the γ-Al_2_O_3_ surface [13,30,35]. The crystallite sizes of MnFe_2_O_4_ and FMA were further estimated using the Scherrer equation based on the (311) diffraction peak [43]. The calculated crystallite size decreased from 32.8 nm for MnFe_2_O_4_ to 8.8 nm for FMA, confirming that γ-Al_2_O_3_ effectively inhibited MnFe_2_O_4_ crystallite growth during the impregnation–calcination process. This result is consistent with the TEM observation, where the average particle size decreased from 35.6 nm to 12.7 nm, further supporting the dispersion-regulation effect of γ-Al_2_O_3_. No additional diffraction peaks associated with impurity phases are detected, confirming the successful formation of the supported MnFe_2_O_4_ composite.

**Figure 2:**
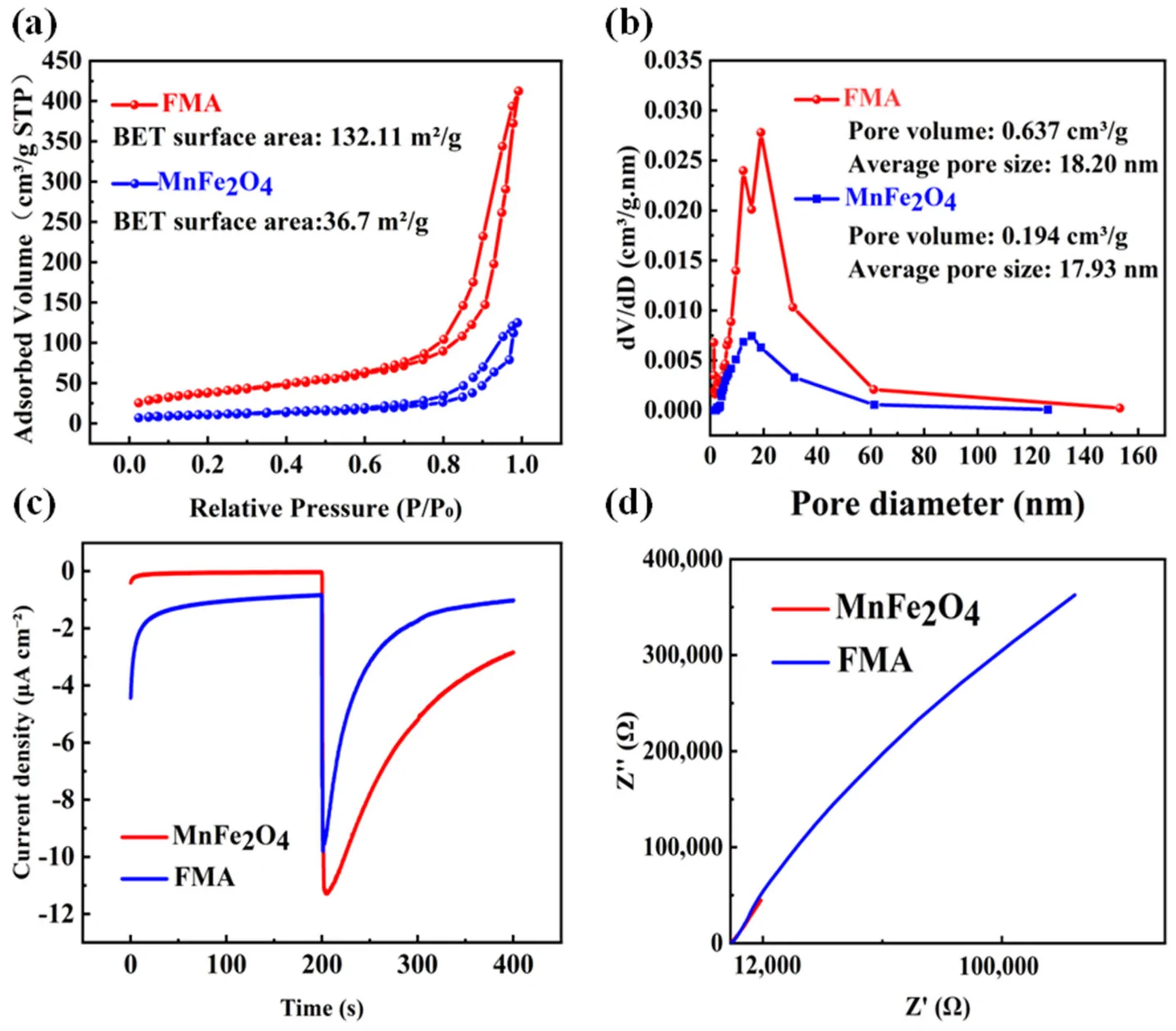
(**a**) N_2_ adsorption–desorption isotherms and (**b**) pore-size distributions of MnFe_2_O_4_ and FMA; (**c**) current–time (i–t) curves of MnFe_2_O_4_ and FMA recorded at 0.00 V; and (**d**) Nyquist plots of MnFe_2_O_4_ and FMA recorded at open-circuit potential. Reaction conditions: Catalyst dosage = 0.5 g L^−1^, T = 80 °C, and [H_2_O_2_]_0_ = 110 mM.

Nitrogen adsorption–desorption measurements were conducted to investigate the porous characteristics and surface accessibility of the catalysts. As shown in Figure 2a,b, both MnFe_2_O_4_ and FMA exhibit type IV adsorption–desorption isotherms with hysteresis loops, indicating the presence of mesoporous structures. Bare MnFe_2_O_4_ possesses a relatively low specific surface area of 36.70 m^2^ g^−1^ and a pore volume of 0.194 cm^3^ g^−1^. In comparison, the pristine γ-Al_2_O_3_ support exhibits a high specific surface area of 146.9 m^2^ g^−1^, with a pore volume of 1.20 cm^3^ g^−1^ and an average pore size of 19.8 nm [44], providing a highly porous framework for MnFe_2_O_4_ dispersion. After integration with γ-Al_2_O_3_, the BET surface area and pore volume increase substantially to 132.11 m^2^ g^−1^ and 0.637 cm^3^ g^−1^, respectively, which are substantially higher than those of bare MnFe_2_O_4_. The slightly decreased textural parameters compared with pristine γ-Al_2_O_3_ are likely associated with the partial occupation of pore channels by MnFe_2_O_4_ nanoparticles, whereas the retained porous structure originates from the intrinsic porosity of γ-Al_2_O_3_ and the improved dispersion of MnFe_2_O_4_ nanoparticles [30,32,35]. Importantly, the resulting porous and accessible interface is expected to increase the exposure of surface Fe/Mn active centers and provide additional transport pathways for PVA chains and H_2_O_2_ [6,14,45]. This structural feature is particularly relevant to concentrated PVA and gel-related polymer networks, in which extensive hydrogen bonding, polymer-chain entanglement, and restricted molecular diffusion can limit reactant transport toward catalytic interfaces. Thus, the γ-Al_2_O_3_-mediated porous architecture provides a structural basis for improving interfacial accessibility and subsequent H_2_O_2_-driven oxidative conversion.

**Figure 3:**
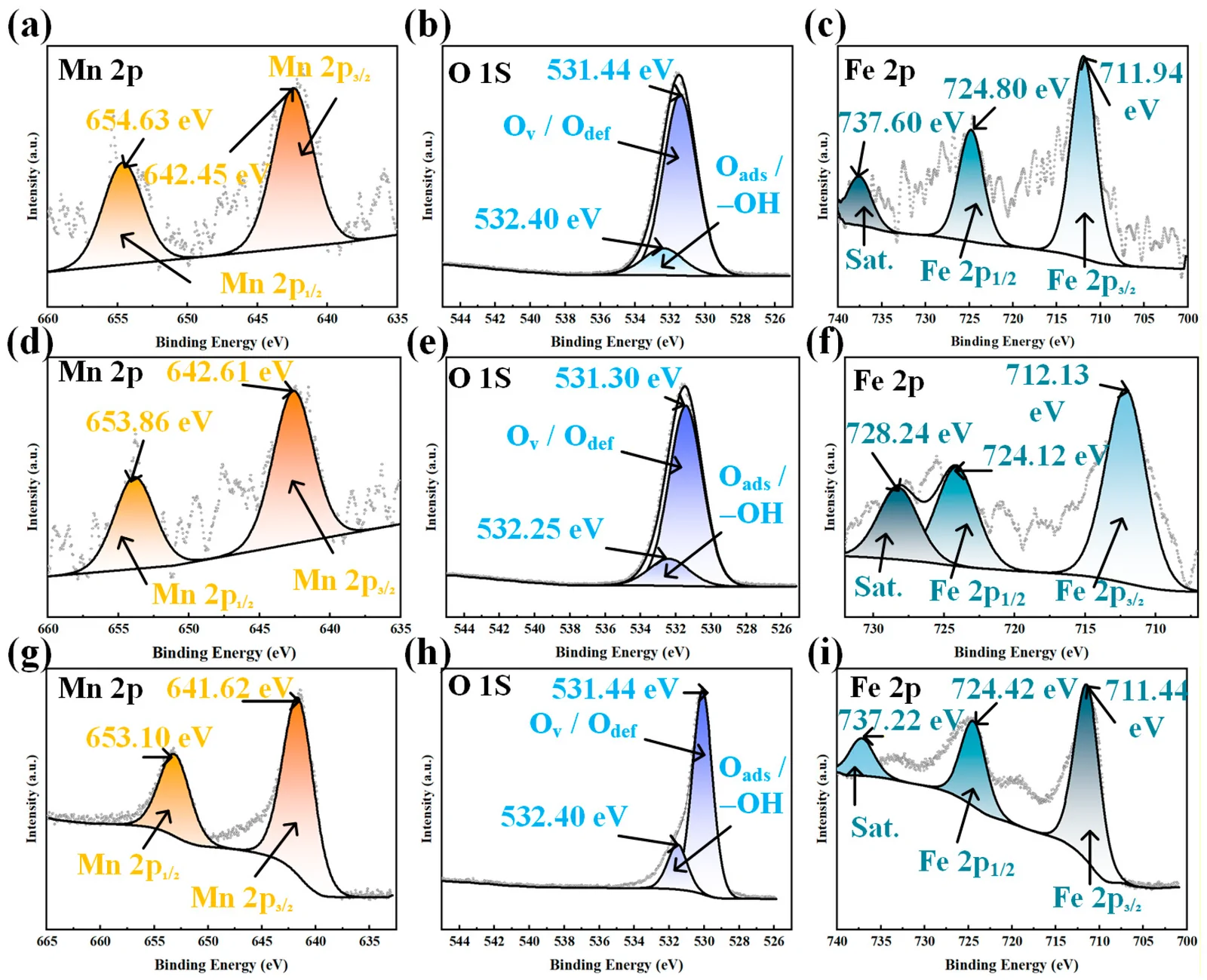
High-resolution XPS spectra of MnFe_2_O_4_, fresh FMA, and used FMA after PVA oxidative conversion: (**a**,**d**,**g**) Mn 2p, (**b**,**e**,**h**) O 1s, and (**c**,**f**,**i**) Fe 2p.

The surface chemical states of Mn species were investigated by high-resolution Mn 2p XPS spectroscopy (Figure 3a,d,g). The characteristic Mn 2p_3/2_ and Mn 2p_1/2_ signals confirm the presence of Mn species in the MnFe_2_O_4_-containing phase. Deconvolution of the Mn 2p_3/2_ region reveals the coexistence of Mn^2+^ and Mn^3+^ species on the catalyst surface [14,27,28], providing multivalent Mn centers capable of participating in surface redox processes. After the catalytic reaction, a slight shift in Mn binding energy together with a change in the relative distribution of Mn oxidation states was observed, suggesting the participation of Mn species in surface electron-transfer processes during H_2_O_2_ activation [14,28,29]. These changes indicate a dynamic redox response of surface Mn centers under reaction conditions and support their involvement in the interfacial redox processes of the FMA/H_2_O_2_ system.

The O 1s spectra (Figure 3b,e,h) were deconvoluted into two main components at approximately 531.3–531.4 and 532.3–532.4 eV, which were assigned to defect-related oxygen species (O_v_/O_def_) and surface-adsorbed oxygen/hydroxyl species (O_ads_/–OH), respectively. Compared with bare MnFe_2_O_4_, fresh FMA exhibited slight shifts of both O 1s components toward lower binding energies, suggesting that the interaction between MnFe_2_O_4_ and γ-Al_2_O_3_ modifies the local electronic environment of surface oxygen species. Such interfacial modulation may contribute to the regulation of surface redox properties and facilitate H_2_O_2_ adsorption and activation. After catalytic reaction, the O 1s peaks shifted slightly back to higher binding energies, indicating dynamic changes in the surface oxygen environment during PVA oxidation. These results suggest that the MnFe_2_O_4_/γ-Al_2_O_3_ interface is associated with changes in the surface chemical environment of the FMA catalyst.

The Fe 2p spectra (Figure 3c,f,i) display the characteristic Fe 2p_3/2_ and Fe 2p_1/2_ signals together with the corresponding satellite features, revealing the coexistence of Fe^2+^ and Fe^3+^ species on the catalyst surface [17,46,47]. The presence of mixed-valence Fe species provides the redox basis for heterogeneous Fenton-like H_2_O_2_ activation [16,17,41,46,47]. After the catalytic reaction, the relative distribution of Fe^2+^ and Fe^3+^ species changes compared with that of fresh FMA, indicating that surface Fe centers undergo redox conversion during PVA oxidation. Such dynamic Fe^2+^/Fe^3+^ conversion is closely associated with continuous H_2_O_2_ activation and the subsequent generation of hydroxyl radicals [14,17,28,47]. Together with the changes observed in the Mn oxidation states, these results support the participation of both Fe and Mn centers in the surface redox processes responsible for H_2_O_2_ activation.

The interaction between MnFe_2_O_4_ and γ-Al_2_O_3_ further influences the local chemical environment of surface Fe/Mn species. Compared with unsupported MnFe_2_O_4_, the Fe and Mn species in FMA exhibited shifted binding energies, suggesting that the MnFe_2_O_4_/γ-Al_2_O_3_ interface influences the local chemical environment of surface Fe/Mn centers [34,38,39,40]. Meanwhile, γ-Al_2_O_3_ effectively suppresses the aggregation of MnFe_2_O_4_ nanoparticles, resulting in smaller particle size and increased accessible surface area. The enhanced dispersion of MnFe_2_O_4_ and the increased exposure of surface-active sites contribute to the improved catalytic performance of FMA. In addition, the interfacial interaction between MnFe_2_O_4_ and γ-Al_2_O_3_ may regulate the electronic structure of Fe/Mn species, thereby facilitating Fe/Mn redox cycling and H_2_O_2_ activation [31,34,36,39,40,46].

**Figure 4:**
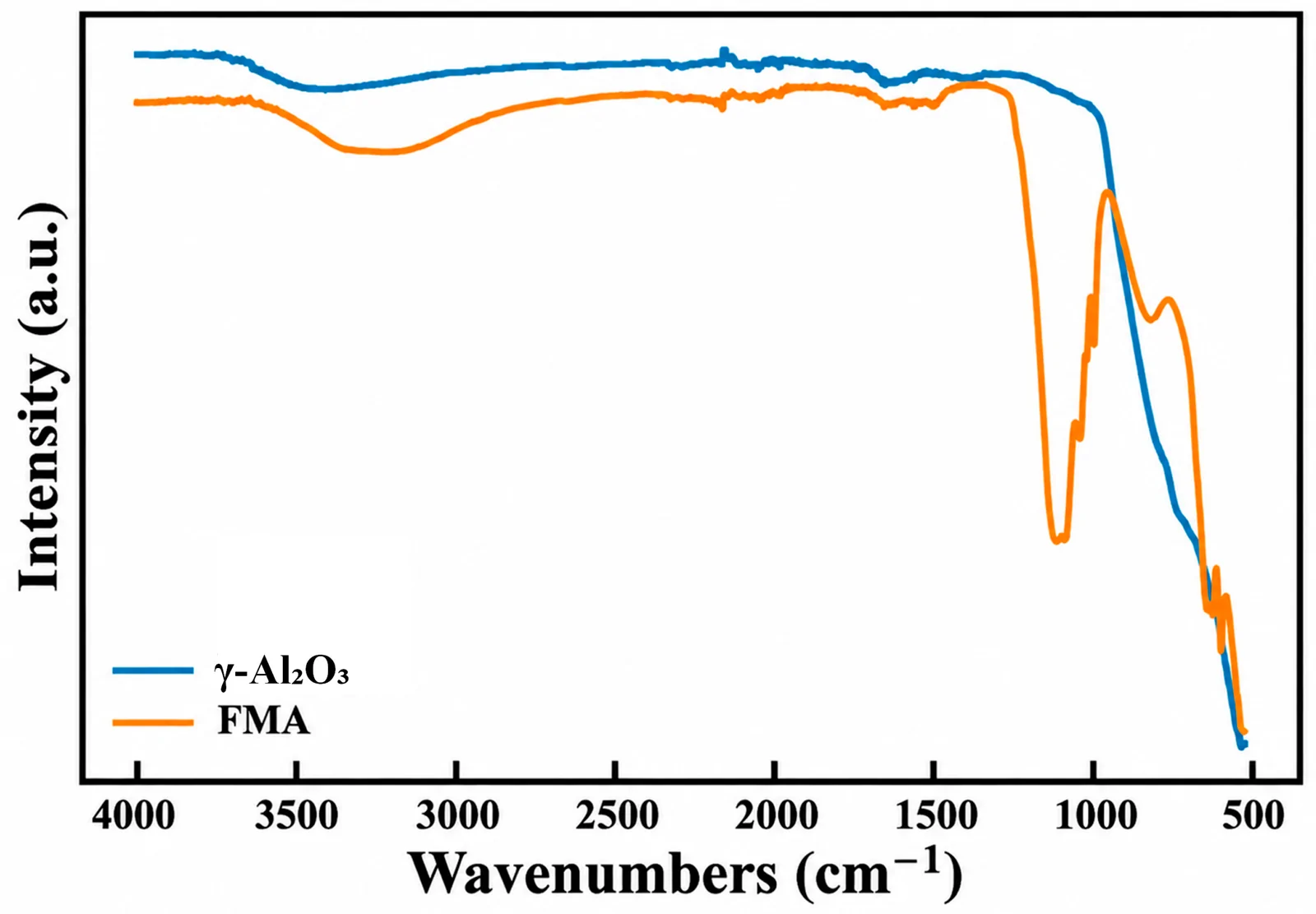
FTIR spectra of γ-Al_2_O_3_ and FMA samples.

The surface functional groups and the interaction between MnFe_2_O_4_ and γ-Al_2_O_3_ were further investigated by FTIR spectroscopy. As shown in Figure 4, γ-Al_2_O_3_ exhibits characteristic hydroxyl-related bands at approximately 3450 cm^−1^ and 1630 cm^−1^, which can be assigned to the stretching vibration of surface hydroxyl groups and the bending vibration of adsorbed water, respectively [48]. The surface hydroxyl groups of γ-Al_2_O_3_ are known to play an important role in the anchoring and dispersion of supported metal species during catalyst preparation [49]. After loading MnFe_2_O_4_, the intensity and position of the hydroxyl-related bands changed compared with pristine γ-Al_2_O_3_, indicating that the local environment of surface hydroxyl groups was modified during MnFe_2_O_4_ formation. Similar variations in hydroxyl bands have been reported for alumina-supported metal oxide catalysts, where the consumption or rearrangement of surface Al–OH groups was associated with the interaction between deposited metal species and alumina supports [50]. These results suggest that surface Al–OH groups participate in the immobilization of Fe/Mn precursor species, thereby facilitating the formation of a highly dispersed MnFe_2_O_4_ phase on γ-Al_2_O_3_. In addition, the characteristic metal–oxygen vibration bands in the low-wavenumber region were retained after MnFe_2_O_4_ loading, confirming the successful incorporation of metal oxide species without destroying the alumina framework [51]. Combined with the XPS results showing changes in the electronic environment of Fe and Mn species, the FTIR results provide complementary evidence for the formation of interfacial interactions between MnFe_2_O_4_ and γ-Al_2_O_3_. Therefore, the enhanced catalytic activity of FMA can be attributed not only to improved MnFe_2_O_4_ dispersion but also to the regulation of Fe/Mn active sites by the γ-Al_2_O_3_ interface.

### 2.2. Identification of Reactive Oxygen Species and Catalytic Stability of FMA

**Figure 5:**
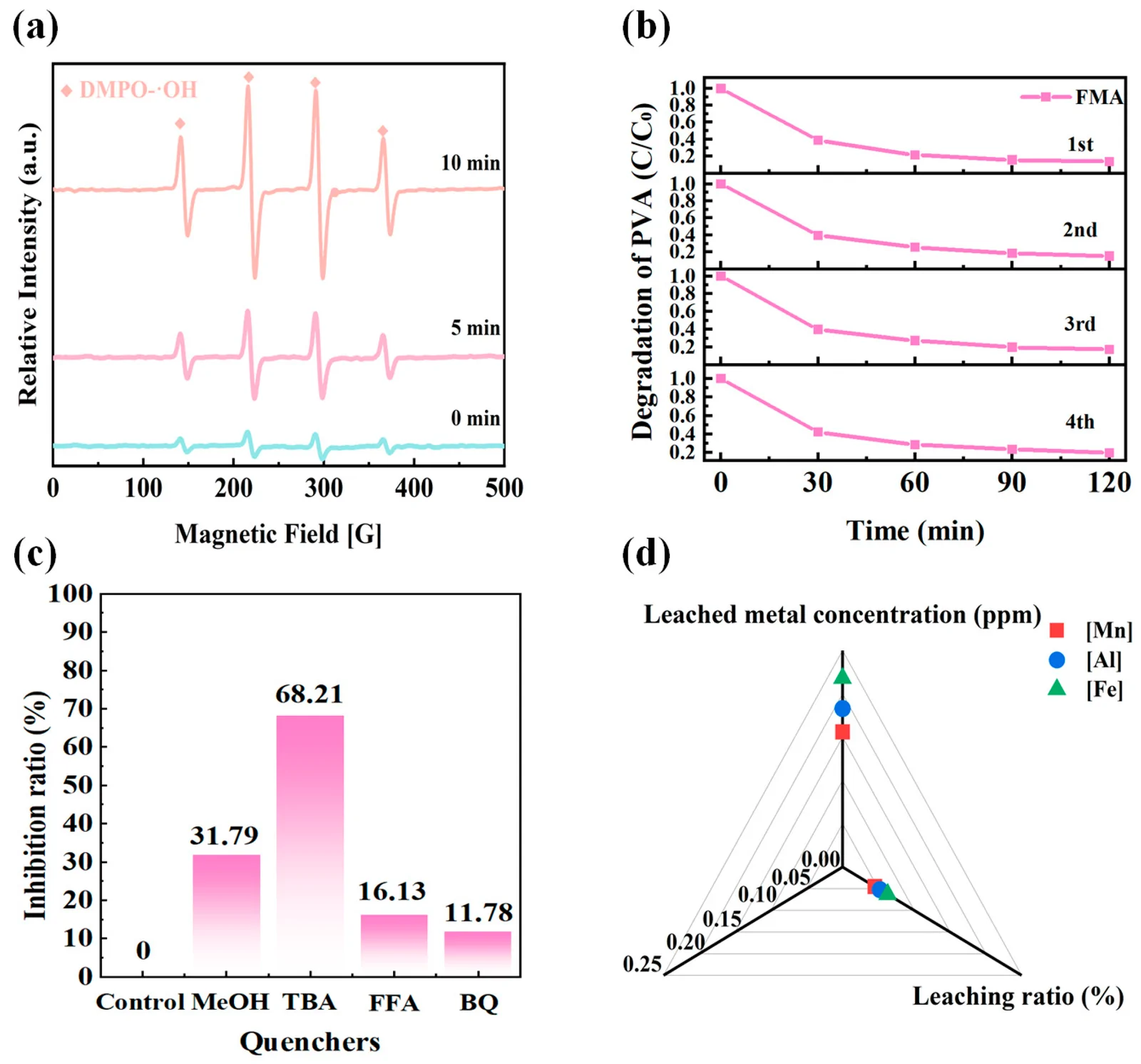
Reactive species identification, catalytic recyclability, and structural stability of the FMA/H_2_O_2_ system. (**a**) DMPO-trapped EPR spectra of ·OH at different reaction times. (**b**) PVA oxidative conversion performance of FMA over four consecutive reuse cycles. (**c**) Radical quenching experiments using MeOH, TBA, FFA and BQ. (**d**) Leached concentrations and corresponding leaching ratios of Mn, Al, and Fe after four consecutive reaction cycles. General condition: C_0_ (Catalyst) = 0.5 g L^−1^, Water content = 90.2%,T = 80 °C, C_0_ (H_2_O_2_) = 110 mM, pH = 3.

Electron paramagnetic resonance (EPR) spectroscopy was employed to identify the reactive oxygen species generated during the FMA/H_2_O_2_ reaction [14,16,17]. As shown in Figure 5a, a characteristic quartet signal with an intensity ratio of 1:2:2:1 was detected after the addition of DMPO, confirming the formation of DMPO–·OH adducts [16,47]. The intensity of the DMPO–·OH signal increased rapidly with reaction time and reached a stable level, indicating continuous hydroxyl radical generation during H_2_O_2_ activation [17,28,47]. The generation of ·OH can be associated with the synergistic redox activity of Fe/Mn centers in the FMA catalyst, where multivalent metal sites facilitate H_2_O_2_ activation and reactive oxygen species formation.

To further verify the contribution of reactive species, radical scavenging experiments were performed using MeOH, TBA, FFA, and BQ. TBA is commonly used as a selective quencher for hydroxyl radicals [16,47], whereas MeOH can react with various oxygen-centered radicals. As shown in Figure 5c, the addition of TBA resulted in the most significant inhibition of PVA degradation, decreasing the degradation efficiency by 68.21%, indicating that ·OH plays a dominant role in the oxidative degradation of PVA. This result is consistent with the characteristic DMPO–·OH EPR signal observed in Figure 5a, further confirming the important contribution of hydroxyl radicals to polymer-chain scission [15,18]. The inhibition effects of MeOH (31.79%), FFA (16.13%), and BQ (11.78%) were relatively weaker, suggesting that other oxygen-centered reactive species, including ^1^O_2_ and O_2_•^−^, may participate in the oxidation process but contribute to a lesser extent. The limited influence of FFA and BQ indicates that these species are not the predominant oxidants in the FMA/H_2_O_2_ system. Therefore, the degradation of PVA mainly proceeds through ·OH-mediated oxidation, accompanied by minor contributions from other reactive oxygen species.

The catalytic stability of FMA was evaluated through consecutive regeneration cycles. After each catalytic reaction, the catalyst was separated, washed thoroughly with deionized water, dried, and calcined at 450 °C before being reused in the next cycle. The catalyst was therefore evaluated under repeated regeneration conditions to assess its stability during long-term catalytic operation. To further evaluate the structural stability of FMA during repeated catalytic operation, the morphology and crystalline structure of the used catalyst after four cycles were examined. As shown in Figure 1d,g, the used FMA maintained a dispersed morphology, and no obvious large-scale aggregation of MnFe_2_O_4_ nanoparticles was observed after four catalytic cycles. In addition, the XRD pattern of the used FMA (Figure 1i) retained the characteristic diffraction peaks of cubic spinel MnFe_2_O_4_, confirming that the crystal structure remained stable after repeated catalytic reactions and calcination treatments. After each reaction, the catalyst was separated, washed, dried, and recycled before reuse. As shown in Figure 5b, FMA retained high catalytic activity after repeated operation, with PVA conversion efficiencies decreasing slightly from approximately 90% in the first cycle to above 85% after four cycles. The slight activity decline may be associated with the partial occupation of surface active sites by oxidation intermediates and minor changes in the catalyst surface during repeated H_2_O_2_ activation [14,40,47]. Nevertheless, the sustained catalytic performance indicates the structural stability of the MnFe_2_O_4_/γ-Al_2_O_3_ interface under oxidative conditions [32,37,40]. The concentrations of Mn, Fe, and Al released into the reaction solution were quantified by ICP-OES (Figure 5d). Only trace amounts of metal ions were detected after repeated reactions, with leaching ratios below 0.1%, indicating effective immobilization of MnFe_2_O_4_ components within the γ-Al_2_O_3_-supported structure.

### 2.3. Synergistic Contribution of Al_2_O_3_-Supported MnFe_2_O_4_ Interface in PVA Oxidation

**Figure 6:**
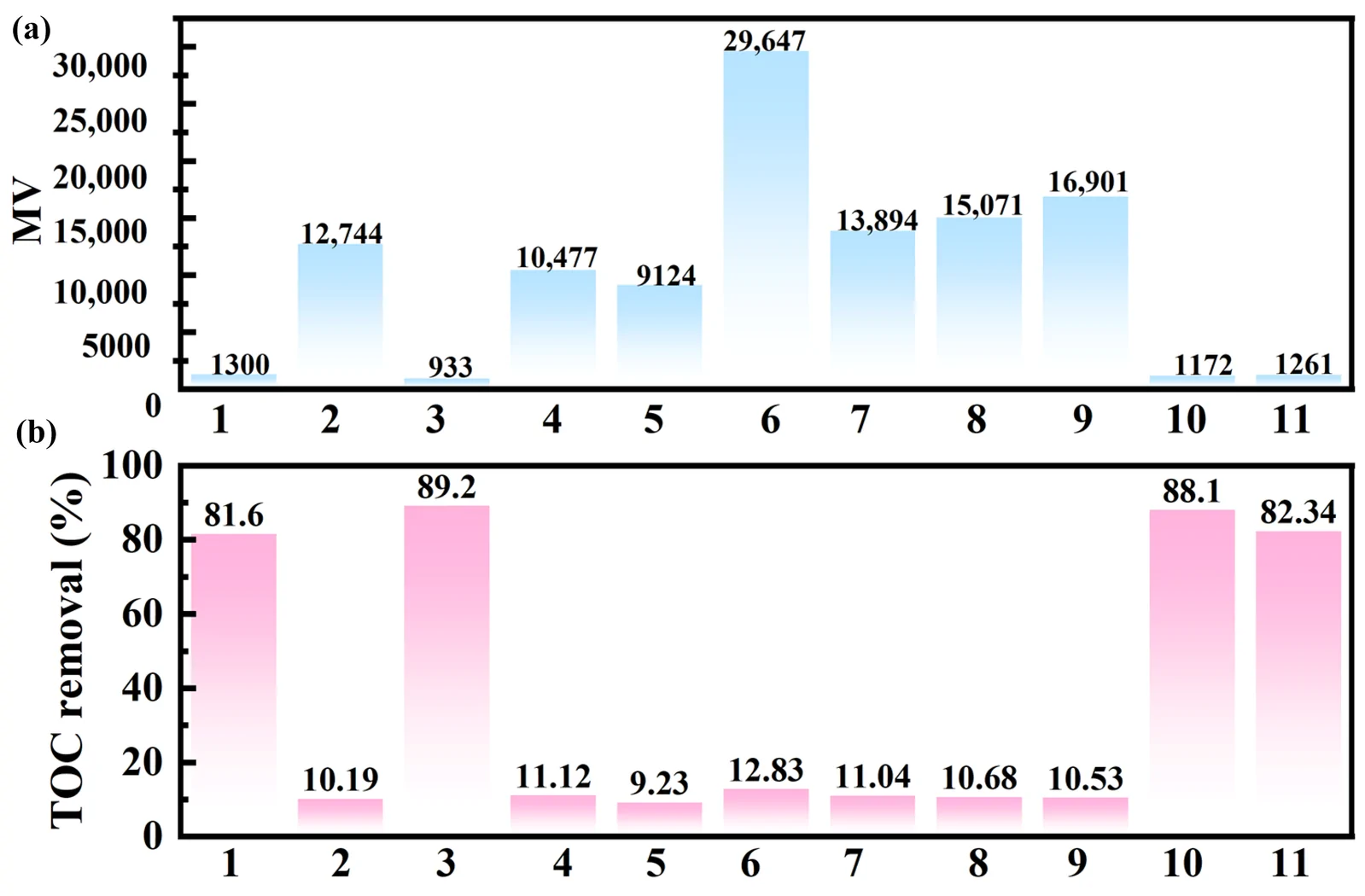
Evolution of PVA degradation and mineralization performance in different catalytic systems. The catalytic systems are denoted as follows: (1) MnFe_2_O_4_ + H_2_O_2_, (2) FMA, (3) FMA + H_2_O_2_, (4) H_2_O_2_, (5) Al_2_O_3_ + Al_2_O_3_, (6) MnFe_2_O_4_, (7) Al_2_O_3_, (8) Fe/Al_2_O_3_, (9) Mn/Al_2_O_3_, (10) Fe/Al_2_O_3_ + H_2_O_2_, and (11) Mn/Al_2_O_3_ + H_2_O_2_. (**a**) Changes in the molecular weight (MV) of PVA after catalytic treatment in different systems. (**b**) TOC removal efficiencies of PVA under different catalytic systems. General condition: C_0_ (Catalyst) = 0.5 g L^−1^, Water content = 90.2%, T = 80 °C, C_0_ (H_2_O_2_) = 110 mM, pH = 3.

The molecular-level characterization of polymer structures is important for understanding degradation behavior. Various spectroscopic approaches have been developed to probe polymer-chain conformation and dynamics [52]. In this work, GPC analysis was employed to evaluate oxidative chain scission by monitoring molecular-weight reduction. GPC and TOC analyses were performed to evaluate PVA chain degradation and mineralization efficiency, respectively. As shown in Figure 6a, H_2_O_2_ alone and individual catalyst systems caused limited molecular-weight reduction, which was consistent with their low TOC removal efficiencies (9.23–12.83%). These results indicate that direct oxidation by H_2_O_2_ or single-component catalysts was insufficient for effective PVA degradation. Compared with Fe/Al_2_O_3_ and Mn/Al_2_O_3_ catalysts, the MnFe_2_O_4_-based system exhibited enhanced degradation and mineralization performance, demonstrating the synergistic effect of Fe/Mn redox couples in H_2_O_2_ activation and ROS generation. In particular, MnFe_2_O_4_/H_2_O_2_ achieved 81.6% TOC removal, while further incorporation of γ-Al_2_O_3_ significantly improved the catalytic efficiency (Figure 6b). The FMA/H_2_O_2_ system showed the greatest molecular-weight decrease and the highest TOC removal (89.2%), which can be attributed to improved MnFe_2_O_4_ dispersion, increased exposure of active sites, and accelerated interfacial H_2_O_2_ activation. Therefore, the MnFe_2_O_4_–γ-Al_2_O_3_ interface effectively integrates Fe/Mn synergistic oxidation and support-assisted dispersion, leading to efficient PVA degradation and mineralization.

### 2.4. Optimization of Reaction Parameters and Interfacial Catalytic Performance Toward Concentrated PVA Gel-Forming Networks

#### 2.4.1. Effect of Catalyst Dosage on PVA Oxidative Conversion

**Figure 7:**
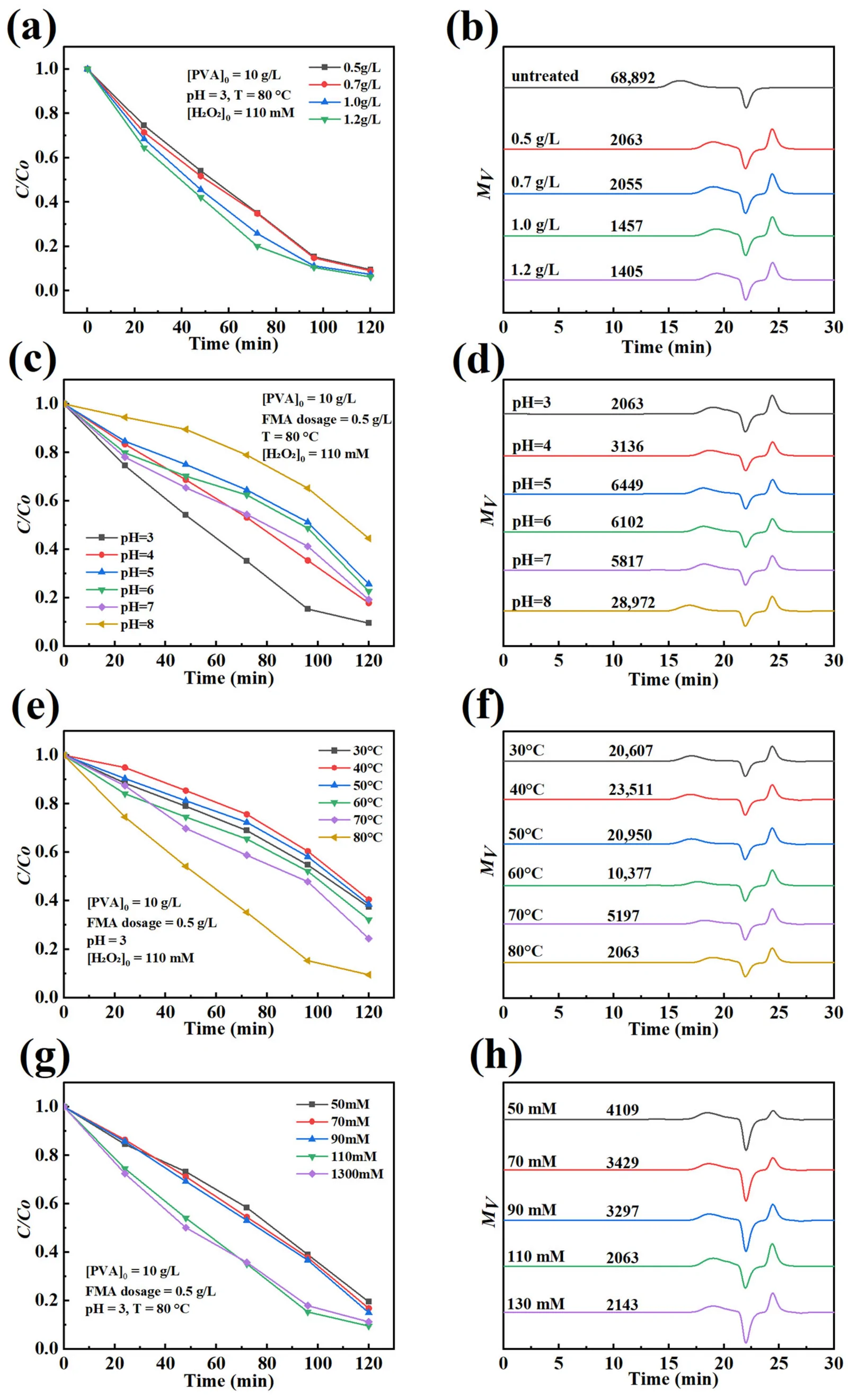
Effects of catalyst dosage (**a**,**b**), pH (**c**,**d**), temperature (**e**,**f**), and H_2_O_2_ concentration (**g**,**h**) on the PVA oxidative conversion. General condition: C_0_ (Catalyst) = 0.5 g L^−1^, Water content = 90.2%, T = 80 °C, C_0_ (H_2_O_2_) = 110 mM, pH = 3.

Figure 7a,b illustrates the influence of FMA dosage on PVA oxidative conversion. As the FMA dosage increased from 0.5 to 1.2 g L^−1^, PVA conversion increased progressively, especially during the initial reaction stage. After 120 min of reaction, the residual PVA concentration fell below 10% under all tested FMA dosages, indicating high catalytic activity of the FMA/H_2_O_2_ system toward concentrated PVA. As the FMA dosage increased, more accessible Fe/Mn redox-active sites became available for H_2_O_2_ activation, thereby promoting reactive oxygen species generation and subsequent oxidative transformation of PVA chains. The possible redox reactions between Fe/Mn active sites and H_2_O_2_ can be described as follows [14,17,28,29]:

(1) $$Fe^{2+}/Mn^{2+}+H_{2}O_{2}\to Fe^{3+}/Mn^{3+}+\cdot \mathrm{OH}+OH^{-}$$

(2) $$Fe^{3+}/Mn^{3+}+H_{2}O_{2}\to Fe^{2+}/Mn^{2+}+HO_{2}\cdot +H^{+}$$

The generated ·OH can subsequently attack PVA chains, leading to oxidative chain scission [15,18,53]. However, the improvement became less pronounced when the FMA dosage exceeded 1.0 g L^−1^, suggesting that excessive FMA did not further enhance PVA conversion significantly. This phenomenon may be associated with the self-quenching of reactive radicals or the limited availability of H_2_O_2_ at high FMA loading [16,17,47]. Excessive metal active sites may consume ·OH according to the following reaction [16]:

(3) $$Fe^{2+}/Mn^{2+}+\cdot \mathrm{OH}\to Fe^{3+}/Mn^{3+}+OH^{-}$$

Consistently, the GPC results in Figure 7b showed an obvious decrease in the molecular weight of PVA from 68,892 Da for untreated PVA to approximately 1400–2100 Da after treatment, demonstrating pronounced polymer-chain scission during the oxidative conversion process. Considering the comparable PVA conversion performance and lower catalyst consumption, 0.5 g L^−1^ was selected as the preferred dosage.

#### 2.4.2. Effect of pH on PVA Oxidative Conversion

Figure 7c,d illustrate the influence of the initial pH on PVA oxidative conversion. The PVA conversion efficiency was found to be highly sensitive to the solution pH. Under acidic conditions, particularly at pH 3, PVA conversion proceeded much more rapidly, and the residual PVA concentration decreased to approximately 10% after 120 min. In contrast, the PVA conversion efficiency was markedly inhibited under alkaline conditions, especially at pH 8, where nearly 45% of PVA remained after 120 min. This result indicates that acidic conditions are more favorable for H_2_O_2_-driven oxidative conversion of PVA in this catalytic system.

The superior PVA conversion performance under acidic conditions may be ascribed to the facilitated activation of H_2_O_2_ and the enhanced formation of highly oxidative radical species [14,17]. Under acidic conditions, Fe/Mn species can efficiently activate H_2_O_2_ to produce ·OH (as shown in Equations (2) and (3)) [17,28,29]. The generated ·OH are highly oxidative and can attack the C–C and C–O bonds of PVA chains, resulting in oxidative polymer-chain scission [15,18,53]. In alkaline media, however, the decomposition of H_2_O_2_ may be accelerated through nonproductive pathways. Meanwhile, excessive OH^-^ may scavenge ·OH and reduce the effective concentration of reactive oxygen species (as shown in Equation (5)) [16,47].

(4) $$\cdot \mathrm{OH}+OH^{-}\to \cdot O_{2}^{-}+H_{2}O$$

These side reactions weaken the oxidation capacity of the catalytic system, leading to a reduced PVA conversion efficiency under alkaline conditions. The molecular weight distribution in Figure 7d further supports this conclusion. The treated PVA showed much lower molecular weights under acidic and neutral conditions, whereas a relatively high molecular weight of 28,972 Da was still observed at pH 8, indicating less effective polymer-chain scission under alkaline conditions. Therefore, pH 3 was selected as the optimum initial pH for PVA oxidative conversion.

#### 2.4.3. Effect of Temperature on PVA Oxidative Conversion

Figure 7e,f presents the effect of reaction temperature on PVA oxidative conversion. The PVA conversion rate increased remarkably with increasing temperature from 30 to 80 °C. At 30–70 °C, PVA conversion increased progressively, whereas the most significant conversion was achieved at 80 °C, with the residual PVA concentration decreasing to below 10% after 120 min. This enhancement could be explained by enhanced molecular transport, increased molecular collision frequency, and more efficient interfacial H_2_O_2_ activation at elevated temperatures, which jointly contributed to the increased production of reactive oxygen species [14,17,22]. Moreover, higher temperature could facilitate the oxidative cleavage of PVA molecular chains, as evidenced by the GPC results in Figure 7f. The molecular weight decreased from 20,607 Da at 30 °C to 2063 Da at 80 °C, indicating more pronounced polymer-chain scission at elevated temperatures. Accordingly, 80 °C was chosen as the optimum reaction temperature.

#### 2.4.4. Effect of H_2_O_2_ Dosage on PVA Oxidative Conversion

The impact of H_2_O_2_ dosage on PVA oxidative conversion was further examined (Figure 7g,h). As the concentration increased from 50 to 110 mM, PVA conversion was significantly enhanced, resulting in a residual concentration of around 10% after 120 min at 110 mM H_2_O_2_. Within this concentration range, more oxidant molecules were available to interact with the accessible Fe/Mn redox-active sites on FMA, thereby promoting reactive oxygen species generation [14,16,17,54] and enhancing the oxidative conversion of PVA.

As illustrated in Equations (2) and (3), the interfacial Fe/Mn redox processes on FMA facilitate H_2_O_2_ activation and promote ·OH generation [14,17,28,29,54]. The resulting radicals then react with PVA chains, thereby inducing oxidative chain scission and forming lower-molecular-weight products. However, when the H_2_O_2_ concentration was further increased to 130 mM, the PVA conversion efficiency did not continue to improve. Instead, a slight decline was observed, indicating that excessive H_2_O_2_ could negatively affect the oxidation process. Although a higher H_2_O_2_ dosage provides more precursor molecules for ROS generation, surplus H_2_O_2_ may also act as a scavenger of hydroxyl radicals, resulting in the consumption of active species and a reduction in effective oxidative capacity [16,17,22]. In addition, excessive H_2_O_2_ can promote radical self-quenching reactions, leading to the formation of less reactive species such as HO_2_· [16,17,22] (as shown in Equations (6) and (7)). These side reactions decrease the availability of ·OH for PVA oxidative conversion and lower the utilization efficiency of H_2_O_2_.

(5) $$H_{2}O_{2}+\cdot \mathrm{OH}\to HO_{2}\cdot +H_{2}O$$

(6) $$HO_{2}\cdot +\cdot \mathrm{OH}\to H_{2}O+O_{2}$$

These radical-consuming reactions explain why excessive H_2_O_2_ did not further improve PVA oxidative conversion. The molecular weight results in Figure 7h showed that the molecular weight of PVA decreased dramatically under all tested H_2_O_2_ concentrations, with the lowest value obtained at 110 mM, indicating the most pronounced polymer-chain scission under this condition. Therefore, 110 mM H_2_O_2_ was regarded as the optimum concentration for PVA oxidative conversion.

Overall, the oxidative conversion of PVA was highly affected by the catalyst dosage, initial pH, reaction temperature, and H_2_O_2_ concentration. The optimum reaction conditions were determined to be a catalyst dosage of 0.5 g L^−1^, an initial pH of 3, a reaction temperature of 80 °C, and an H_2_O_2_ concentration of 110 mM. Under these conditions, PVA was efficiently converted, and its molecular weight was substantially reduced, demonstrating the effectiveness of the FMA/H_2_O_2_ catalytic system in coupling PVA conversion with pronounced polymer-chain scission.

### 2.5. Proposed Interfacial H_2_O_2_ Activation Mechanism and Oxidative Chain-Scission Pathway of PVA Gel-Forming Networks

**Figure 8:**
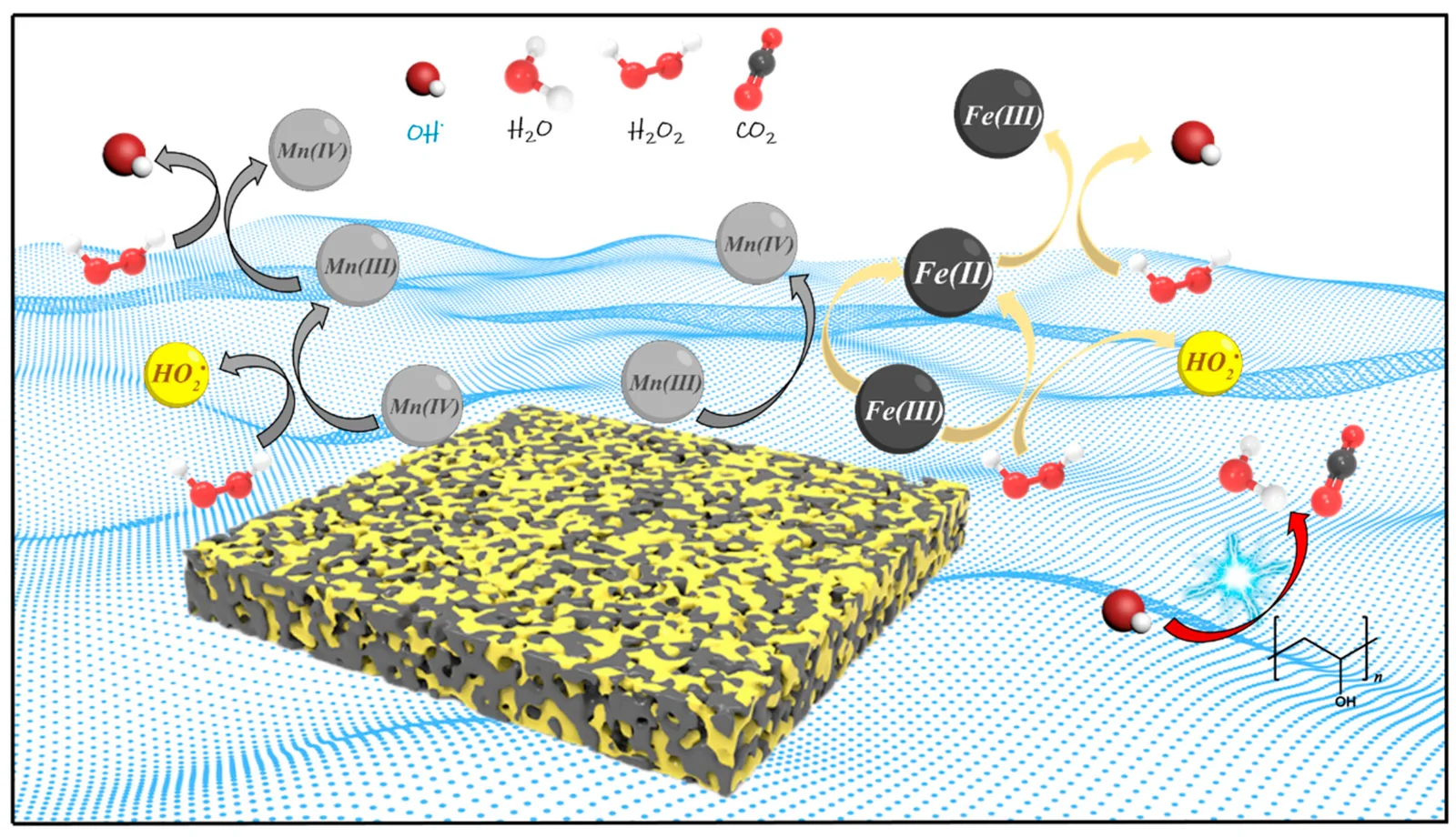
Proposed mechanism of interfacial H_2_O_2_ activation by FMA and subsequent oxidative chain scission of concentrated PVA gel-forming networks.

The proposed activation mechanism is illustrated in Figure 8. The enhanced catalytic activity of FMA can be attributed to the coupling between MnFe_2_O_4_/γ-Al_2_O_3_ interfacial regulation and Fe/Mn redox processes [14,28,35,40]. During the impregnation–calcination process, γ-Al_2_O_3_ provides anchoring sites through surface hydroxyl groups, which suppress the migration and aggregation of MnFe_2_O_4_ nanoparticles and promote the formation of highly dispersed and accessible catalytic domains [33,35,37].

Fe species are generally considered the primary active centers for heterogeneous Fenton-like reactions [23,46]. Surface Fe^2+^ species can react with H_2_O_2_ to generate hydroxyl radicals, accompanied by the formation of Fe^3+^ species (Equation (2)). The regeneration of Fe^2+^ from Fe^3+^ is essential for sustaining catalytic oxidation. In the FMA system, the coexistence of Mn species may facilitate interfacial redox conversion and Fe^2+^ regeneration, thereby maintaining continuous H_2_O_2_ activation [27,28,29,55].

Mn species with variable oxidation states provide additional interfacial redox pathways at the catalyst interface [25,28,55]. The reversible transformation between Mn oxidation states can modulate the local electronic environment and facilitate Fe redox conversion, contributing to sustained ROS generation during H_2_O_2_ activation [14,28,29,55].

The role of γ-Al_2_O_3_ extends beyond providing surface area alone. The porous support regulates the spatial distribution of MnFe_2_O_4_ nanoparticles and improves the accessibility of Fe/Mn active sites [13,32,33,35]. The intimate contact between MnFe_2_O_4_ and γ-Al_2_O_3_ creates abundant and accessible MnFe_2_O_4_/γ-Al_2_O_3_ catalytic interfaces, facilitating H_2_O_2_ adsorption and activation as well as effective contact between the generated ROS and PVA chains.

The generated ·OH radicals possess strong oxidative capability and can attack the carbon backbone and hydroxyl groups of PVA. Hydrogen abstraction and oxygen incorporation reactions can lead to the formation of carbonyl-containing intermediates, followed by cleavage of C–C and C–O bonds [5,18,53]. Consequently, the high-molecular-weight PVA chains are transformed into lower-molecular-weight fragments and oxygenated intermediates, providing a molecular-level pathway for oxidative chain scission. From the perspective of gel-network transformation, the pronounced decrease in PVA molecular weight is expected to progressively weaken the entangled and hydrogen-bonded polymer structure, thereby facilitating the conversion of highly associated gel-forming PVA networks into lower-molecular-weight soluble fragments.

### 2.6. Identification of Oxidation Intermediates and Proposed Oxidative Chain-Scission Pathway of PVA Gel-Forming Networks

**Figure 9:**
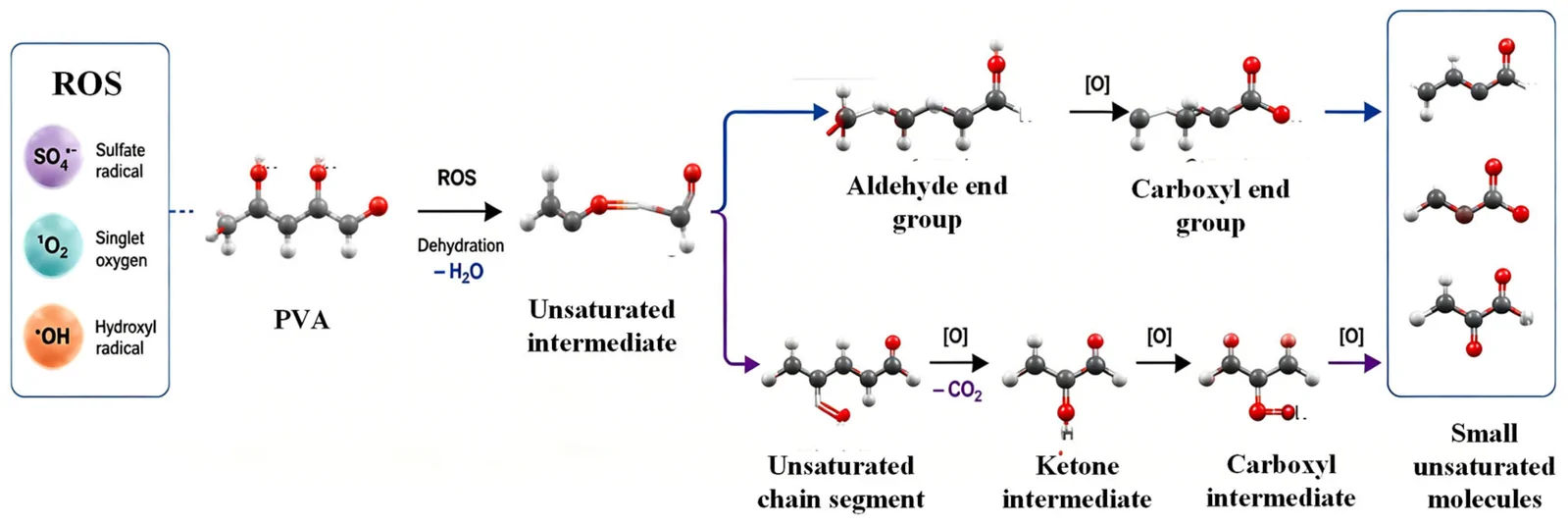
Possible degradation pathways of PVA.

GC-MS analysis was performed to identify soluble oxidation intermediates generated during PVA oxidative conversion. As shown in Figure 9, several oxygen-containing low-molecular-weight compounds were detected after catalytic oxidation, indicating the progressive oxidative transformation of PVA chains. The formation of carbonyl- and carboxyl-containing products suggests that hydroxyl radicals generated at the FMA/H_2_O_2_ catalytic interface participate in hydrogen-abstraction and oxygen-incorporation reactions along the PVA backbone [18,53]. During oxidation, ·OH radicals can attack the carbon backbone and hydroxyl-containing sites of PVA molecules [3,18,53]. The initial oxidation process leads to the formation of oxygenated functional groups, including carbonyl and carboxyl species. Subsequent oxidative cleavage of C–C bonds generates shorter-chain fragments and low-molecular-weight intermediates. Together with the pronounced decrease in PVA molecular weight, these results indicate that oxidative chain scission is a major transformation pathway in the FMA/H_2_O_2_ system within the investigated reaction time [3,5,18]. This behavior is advantageous for the oxidative transformation and deconstruction of concentrated gel-related polymer systems, because rapid molecular-weight reduction can weaken highly entangled PVA networks and improve the downstream processability of the resulting lower-molecular-weight products [3,5]. The above findings also provide insights into the potential applicability of the FMA/H_2_O_2_ system toward other polymeric materials, where degradation efficiency is expected to be strongly governed by polymer structure and accessibility of reactive species [56,57,58]. The FMA/H_2_O_2_ system may therefore have potential for oxidative treatment of other synthetic and natural polymer networks, particularly those containing hydrophilic and oxidation-susceptible functional groups. The effective degradation of PVA is mainly associated with its hydroxyl-rich and water-compatible structure, which facilitates contact with reactive species and promotes ·OH-induced cleavage of C–C and C–O bonds [8,59]. For other polymers, the oxidation efficiency may vary depending on their chemical structures, including hydrophilicity, functional-group reactivity, crystallinity, and crosslinking density, which collectively affect reactive-species accessibility and oxidation susceptibility [60,61].

## 3. Conclusions

A γ-Al_2_O_3_-supported MnFe_2_O_4_ catalyst (FMA) was successfully constructed for H_2_O_2_ activation and oxidative chain scission of concentrated PVA gel-forming networks. The introduction of γ-Al_2_O_3_ effectively suppressed MnFe_2_O_4_ aggregation and improved the accessibility of surface Fe/Mn active sites, resulting in enhanced interfacial catalytic performance. Compared with bare MnFe_2_O_4_, FMA exhibited improved textural properties, with the BET surface area increasing from 36.70 to 132.11 m^2^ g^-1^. The enhanced catalytic performance of FMA originates from the MnFe_2_O_4_/γ-Al_2_O_3_ interfacial synergy, where Fe/Mn redox cycling promotes H_2_O_2_ activation and ·OH-dominated oxidative chain scission of PVA, with minor contributions from other reactive species. Under optimized conditions, approximately 90% PVA conversion was achieved within 120 min, with the molecular weight decreasing from 68,892 to 2063 Da and TOC removal reaching 89.2%, confirming efficient PVA chain scission and mineralization. Furthermore, FMA maintained stable catalytic performance during repeated cycles with negligible metal leaching. This work highlights the potential of the FMA/H_2_O_2_ system as an interfacial oxidation platform for polymer degradation, while emphasizing the critical role of polymer structure in determining oxidation susceptibility. Overall, this work demonstrates that support-mediated interfacial regulation of transition-metal oxides provides an effective strategy for enhancing active-site accessibility, redox functionality, and catalytic efficiency in heterogeneous oxidation systems for the molecular deconstruction of highly entangled, gel-related polymer networks.

## 4. Materials and Methods

### 4.1. Chemicals

Poly(vinyl alcohol) (PVA; degree of polymerization: 2200; degree of hydrolysis: 88 mol%; viscosity: 40–55 mPa·s for a 4 wt% aqueous solution at 20 °C; purity ≥ 99.0%) was purchased from Yongan City Baohua Lin Industrial Development Co., Ltd. (China) Yongan City Baohua Lin Industrial Development Co., Ltd., Yongan, China. A model PVA solution containing 100 g L^−1^ PVA was prepared for all oxidation experiments. The water content of the model PVA system was calculated based on the initial composition and the measured solution density. A PVA solution containing 100 g L^−1^ PVA was prepared using deionized water, with a measured density of 1.02 g mL^−1^. Accordingly, the water mass fraction was calculated to be approximately 90.2 wt%. The PVA concentration and corresponding water content were kept constant for all oxidation experiments. Manganese sulfate monohydrate (MnSO_4_·H_2_O CAS No. 10034-96-5), anhydrous iron(III) sulfate (Fe_2_(SO_4_)_3_ CAS No. 10028-22-5), manganese(II) chloride tetrahydrate (MnCl_2_·4H_2_O CAS No. 13446-34-9), ferric chloride hexahydrate (FeCl_3_·6H_2_O CAS No. 10025-77-1), hydrogen peroxide (H_2_O_2_, CAS No. 7722-84-1 30 wt%), acetone (CAS No. 67-64-1), boric acid (CAS No. 10043-35-3), hydrochloric acid (HCl CAS No. 7647-01-0), and sodium hydroxide (NaOH CAS No. 1310-73-2) were purchased from China National Pharmaceutical Group Corporation China National Pharmaceutical Group Corporation, Beijing, China. Methanol (MeOH CAS No. 67-56-1), γ-crystalline pattern (γ-Al_2_O_3_, φ = 20 nm) (CAS No. 1344-28-1), iodine (CAS No. 7553-56-2 99.9%), tert-butyl alcohol (TBA CAS No. 75-65-0), furfuryl alcohol (FFA CAS No. 98-00-0), 1,4-benzoquinone (BQ CAS No. 106-51-4) and potassium iodide (KI, CAS No. 7681-11-0 99.5%) were supplied by Shanghai Vita Chemical Reagent Co., Ltd. (Shanghai, China). The PVA knitted fabric was used to prepare the concentrated model PVA solution. Unless otherwise specified, all reagents were of analytical grade and used as received without further purification. Deionized water was used throughout the experiments.

### 4.2. Catalyst Preparation

**Scheme 2:**
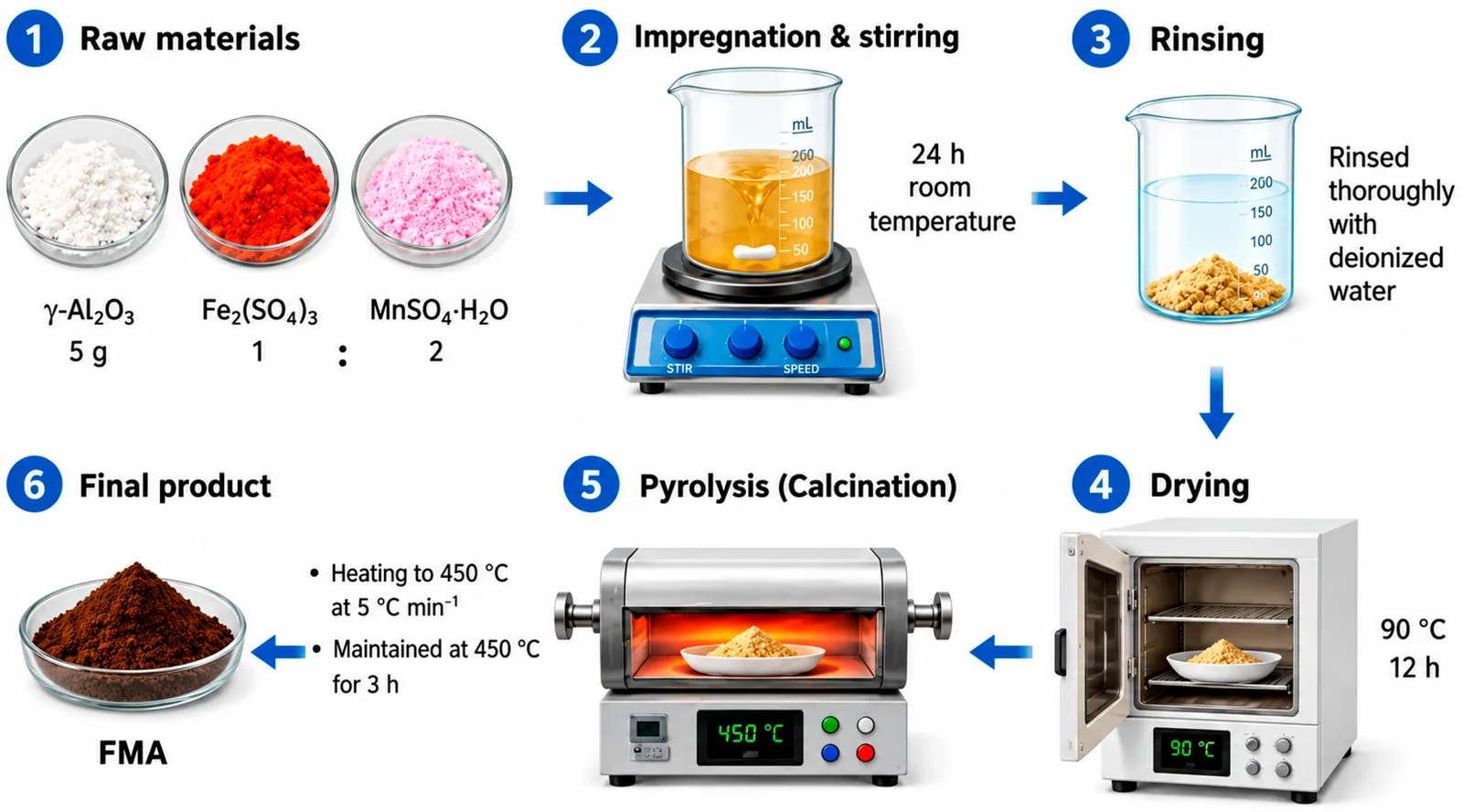
Schematic illustration of the preparation of the FMA catalyst through impregnation and calcination.

The γ-Al_2_O_3_-supported MnFe_2_O_4_ catalyst, denoted as FMA, was synthesized through an impregnation–calcination strategy. Briefly, 5.0 g of γ-Al_2_O_3_ was dispersed in 50 mL of deionized water containing stoichiometric amounts of MnSO_4_·H_2_O and Fe_2_(SO_4_)_3_ at an Mn/Fe molar ratio of 1:2. The suspension was continuously stirred for 24 h at room temperature to promote interaction between the metal precursor species and the surface hydroxyl groups of γ-Al_2_O_3_. The solid was subsequently collected by filtration, washed with deionized water, and dried at 90 °C for 12 h. The obtained precursor was calcined in air at 450 °C for 3 h using a heating rate of 5 °C min^−1^, resulting in MnFe_2_O_4_-containing domains supported on the γ-Al_2_O_3_ surface (Scheme 2). For comparison, bare MnFe_2_O_4_ was prepared using the corresponding procedure in the absence of γ-Al_2_O_3_.

**Scheme 3:**
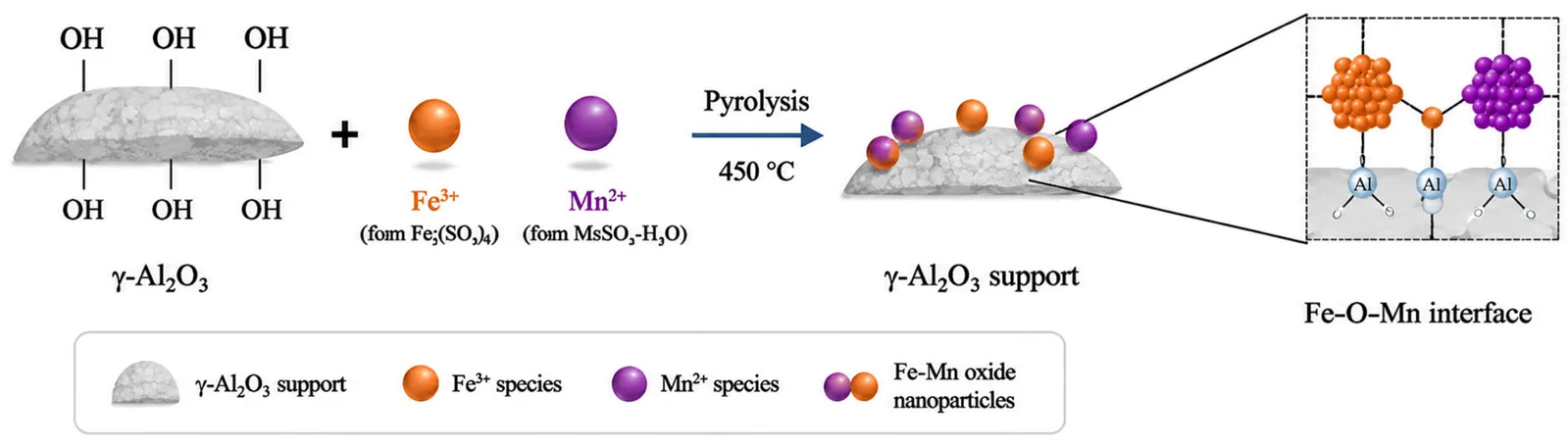
Proposed γ-Al_2_O_3_-mediated formation of spatially dispersed Fe–Mn oxide domains and MnFe_2_O_4_/MnFe_2_O_4_ interfacial structures during impregnation–calcination.

Scheme 3 illustrates the proposed role of γ-Al_2_O_3_ during the impregnation–calcination process. Surface hydroxyl groups on γ-Al_2_O_3_ provide anchoring sites for Fe and Mn precursor species during impregnation, thereby restricting their local migration and aggregation during subsequent thermal treatment [33,35,37]. Calcination converts the supported precursors into spatially dispersed Fe–Mn oxide domains on the γ-Al_2_O_3_ surface, increasing the exposure of Fe/Mn-containing interfacial sites [32,35,41]. The resulting close contact among Fe- and Mn-containing oxide domains and the γ-Al_2_O_3_ support is schematically represented as an interfacial Fe/Mn oxide configuration in Scheme 3. This structural model describes the proposed support-mediated dispersion and interface-formation process and is further evaluated by the structural and surface analyses presented below.

### 4.3. Oxidative Conversion Experiments

For a typical oxidative conversion experiment, 100 mL of the prepared PVA solution was transferred to the reaction vessel, followed by the addition of a predetermined amount of FMA. The suspension was stirred for 30 min before the addition of H_2_O_2_ to establish adsorption–desorption equilibrium between PVA and the catalyst surface. H_2_O_2_ was then added to initiate the oxidation reaction. Unless otherwise specified for the individual parameter investigated, a reaction temperature of 70 °C was used during the initial condition-screening experiments. The effects of FMA dosage, initial solution pH, reaction temperature, and initial H_2_O_2_ concentration on PVA oxidative conversion were systematically investigated by varying one parameter while maintaining the remaining conditions constant.

After favorable operating conditions had been identified, subsequent mechanistic and stability experiments were conducted under the corresponding optimized conditions. Unless otherwise stated, the conditions used for reactive-species identification and related mechanistic experiments were an FMA dosage of 0.5 g L^−1^, an initial pH of 3, an initial H_2_O_2_ concentration of 110 mM, and a reaction temperature of 80 °C.

Control experiments were performed under otherwise identical reaction conditions using FMA + H_2_O_2_, MnFe_2_O_4_ + H_2_O_2_, γ-Al_2_O_3_ + H_2_O_2_, H_2_O_2_ alone, FMA alone, MnFe_2_O_4_ alone, and γ-Al_2_O_3_ alone. These experiments were used to distinguish the catalytic contribution of the supported Fe–Mn oxide phase from those of H_2_O_2_, γ-Al_2_O_3_, and unsupported MnFe_2_O_4_.

For regeneration experiments, the FMA catalyst was recovered after each reaction cycle, washed three times with deionized water, dried overnight at 80 °C, and subsequently calcined at 450 °C for 3 h before reuse. This regeneration procedure was repeated for four consecutive catalytic cycles. Accordingly, the cycling results are referred to herein as regeneration/recycling performance rather than simple catalyst reusability.

### 4.4. Analytical Methods

The morphologies and elemental distributions of the catalysts were examined by scanning electron microscopy (SEM; Quanta F250, FEISEM; Quanta F250, FEI, Hillsboro, OR, USA) equipped with an energy-dispersive X-ray spectrometer (EDS; EDAX GENESIS EDS; EDAX GENESIS, EDAX Inc., Mahwah, NJ, USA). Transmission electron microscopy (TEM) images were acquired using a Tecnai G2 F20 transmission electron microscope (FEI, Hillsboro, OR, USA) operated at 200 kV. The crystalline phases were characterized by X-ray diffraction (XRD) using Cu Kα radiation (λ = 1.5418 Å) over a 2θ range of 10–90°. A TOC-L analyzer (Shimadzu, JapanShimadzu Corporation, Kyoto, Japan) was applied to measure the total organic carbon (TOC). The chemical analysis of the catalysts was carried out by ICP-OES using Varian 715-ES equipment ((ICP-OES; Varian 715-ES, Varian Inc., Palo Alto, CA, USA)).

The textural properties of the catalysts were evaluated by N_2_ adsorption–desorption measurements. The specific surface area was calculated using the Brunauer–Emmett–Teller (BET) method, while the pore-size distribution and pore volume were determined from the adsorption–desorption data. The total pore volume was obtained from the amount of adsorbed nitrogen at P/P_0_ = 0.995.

X-ray photoelectron spectroscopy (XPS; Kratos Axis Ultra XPS; Kratos Axis Ultra, Kratos Analytical Ltd., Manchester, UK) with monochromatic Al Kα radiation was used to analyze the surface chemical states of Mn, Fe, and O species. The binding energies were calibrated using the C 1s peak at 284.8 eV. Fourier-transform infrared (FTIR) spectra were recorded using a Nicolet iS10 spectrometer (Thermo Fisher Scientific, China Thermo Fisher Scientific, Waltham, MA, USA) with KBr pellets to characterize surface functional groups and bonding environments.

The molecular-weight distribution of PVA before and after oxidative treatment was determined by gel permeation chromatography (GPC). The number-average molecular weight (M_n_), weight-average molecular weight (Mw), and polydispersity index (PDI) were obtained from the corresponding molecular-weight distributions.

Residual PVA concentration was quantified by the iodine–boric acid colorimetric method using UV–vis spectroscopy. Absorbance was recorded at 690 nm using a JASCO V-630 UV–vis spectrophotometer (JASCO Corporation, Tokyo, Japan), and PVA concentrations were calculated from calibration curves established under the corresponding pH conditions [62].

The efficiency of PVA oxidative conversion (E) in the FMA+ H_2_O_2_ process was defined as:

(7) $$E\left(\%\right)=\frac{C_{0}-C}{C_{0}}\times 100\%$$

where C_0_ signifies the initial concentration of PVA and C is the concentration of PVA at time at a given reaction time. An increase in the E value reflects an improvement in PVA oxidative conversion efficiency. The degradation behavior of PVA in aqueous solution using the FMA/oxidant process was therefore assessed by varying key operational parameters, namely reaction temperature, H_2_O_2_ concentration, catalyst dosage, and initial solution pH.

## Figures and Tables

**Table 1:** Chemical composition of FMA catalyst determined by ICP-OES.

Catalyst	Mn wt.%	Fe wt.%	Fe/Mn
FMA	4.43	9.42	2.12:1

## Data Availability

The raw data supporting the conclusions of this article will be made available by the authors on request.
